# Rapid Movement of Palmitoleic Acid from Phosphatidylcholine to Phosphatidylinositol in Activated Human Monocytes

**DOI:** 10.3390/biom14060707

**Published:** 2024-06-15

**Authors:** Miguel A. Bermúdez, Alvaro Garrido, Laura Pereira, Teresa Garrido, María A. Balboa, Jesús Balsinde

**Affiliations:** 1 Bioactive Lipids and Lipidomics Core, IBGM, CSIC-UVA, 47003 Valladolid, Spain; 2 Centro de Investigación Biomédica en Red de Diabetes y Enfermedades Metabólicas Asociadas (CIBERDEM), Instituto de Salud Carlos III, 28029 Madrid, Spain; 3 Lipid Metabolism and Inflammation Group, IBGM, CSIC-UVA, 47003 Valladolid, Spain

**Keywords:** palmitoleic acid, phospholipid remodeling, phospholipase A_2_, inflammation, monocytes

## Abstract

This work describes a novel route for phospholipid fatty acid remodeling involving the monounsaturated fatty acid palmitoleic acid. When administered to human monocytes, palmitoleic acid rapidly incorporates into membrane phospholipids, notably into phosphatidylcholine (PC). In resting cells, palmitoleic acid remains within the phospholipid pools where it was initially incorporated, showing no further movement. However, stimulation of the human monocytes with either receptor-directed (opsonized zymosan) or soluble (calcium ionophore A23187) agonists results in the rapid transfer of palmitoleic acid moieties from PC to phosphatidylinositol (PI). This is due to the activation of a coenzyme A-dependent remodeling route involving two different phospholipase A_2_ enzymes that act on different substrates to generate free palmitoleic acid and lysoPI acceptors. The stimulated enrichment of specific PI molecular species with palmitoleic acid unveils a hitherto-unrecognized pathway for lipid turnover in human monocytes which may play a role in regulating lipid signaling during innate immune activation.

## 1. Introduction

The escalating prevalence of diabetes and related metabolic conditions constitutes a pressing concern. Avoiding excess weight appears to be a predominant strategy [1,2,3]. Obesity-induced diabetes often leads to cardiovascular complications, including heart attack and stroke, which involve, to a significant extent, the altered functioning of monocytes and macrophages. Obesity triggers systemic, low-grade inflammation characterized by the elevated levels of immune cells, predominantly macrophages, in metabolic tissues like the skeletal muscle, adipose, and liver. Thus, the accumulation of pro-inflammatory monocyte-derived macrophages in tissues stands as a hallmark of metabolic inflammation in obesity [4,5,6,7,8,9,10,11].

Given their pivotal roles in both physiology and pathophysiology, monounsaturated fatty acids are emerging as significant health indicators. Oleic acid (*cis*-9-octadecenoic acid, 18:1n–9), the predominant monounsaturated fatty acid in human diets, is particularly abundant in olive oil, constituting nearly 80% of its total fatty acid content [12,13]. The Mediterranean diet, renowned for its health-promoting effects and cardio-protective benefits against diseases like cardiovascular diseases, diabetes, and obesity, features a high consumption of olive oil [14,15].

Recent research has brought another monounsaturated fatty acid, palmitoleic acid (*cis*-9-hexadecenoic acid, 16:1n–7, POA), into the limelight of inflammatory lipid research due to its protective effects against hepatic steatosis and its role in enhancing insulin signaling in murine models of metabolic disorders [16,17]. Elevated levels of POA in humans have been shown to correlate with improved insulin sensitivity, a favorable lipid profile, and the reduced incidence of type-2 diabetes and cardiovascular diseases such as myocardial infarction [18]. Additionally, POA reduces inflammation by decreasing circulating pro-inflammatory cytokines and inflammatory markers in various tissues [19,20,21,22,23,24]. This anti-inflammatory activity of POA is quite significant when assessed in cells of the innate immune system, where it is generally comparable to that of omega-3 fatty acids [25,26].

POA can be obtained from dietary sources such as macadamia nuts, dairy, and certain fish. It can also be synthesized via the desaturation of palmitic acid (hexadecanoic acid, 16:0), primarily in adipose tissue and the liver, by stearoyl-CoA desaturase-1, a Δ9 desaturase enzyme [27,28]. Likewise, the incorporation and distribution of POA into the major classes of cellular lipids have recently been characterized in macrophages [26,29]. Results from these studies have shown that in resting phagocytic cells, which generally exhibit low levels of neutral lipids, POA tends to accumulate in membrane phospholipids, particularly phosphatidylcholine (PC) [26,29]. Conversely, in cells with abundant lipid droplets, significant POA localization occurs in these organelles, suggesting a pivotal role of the fatty acid in their metabolism [25,30]. In this study, we have examined the turnover of POA-containing phospholipids in stimulated human monocytes. Our previous work using murine peritoneal macrophages suggested that low POA levels can be mobilized from membrane phospholipids to form POA-containing branched fatty acid esters of hydroxy fatty acids [29], a family of compounds known for their potent anti-inflammatory activity in vivo [31,32]. Our study expands on these findings by utilizing distinct strategies to label the various phospholipid pools. The data suggest that POA is rapidly shuttled between specific phospholipid pools during monocyte activation. This novel lipid remodeling pathway may be instrumental in the execution of specific responses by the activated monocytes.

## 2. Materials and Methods

### 2.1. Reagents

Organic solvents (Optima^®^ LC-MS grade) were from Fisher Scientific (Madrid, Spain). Lipid standards were from Cayman (Ann Arbor, MI, USA) or Avanti (Alabaster, AL, USA). Silicagel G thin-layer chromatography plates were from Macherey-Nagel (Düren, Germany). Additionally, [9,10-^3^H]POA (sp. act. 30–60 Ci/mmol) was from American Radiolabeled Chemicals (St. Louis, MO, USA). The group IVA phospholipase A_2_ (cytosolic phospholipase A_2_α; cPLA_2_α) inhibitor pyrrophenone [33] and the CoA-independent transacylase inhibitor SKF98625 [34] were synthesized and provided by Dr. Alfonso Pérez (Department of Organic Chemistry, University of Valladolid). The group VIA phospholipase A_2_ (calcium-independent phospholipase A_2_β; iPLA_2_β) inhibitors FKGK18 and BEL, and the secreted phospholipase A_2_ (sPLA_2_) inhibitor LY311727, were from Cayman. All other reagents were purchased from Sigma-Aldrich (Madrid, Spain). 

### 2.2. Cell Isolation and Culture

Human peripheral blood monocytes were obtained from buffy coats of healthy anonymous volunteer donors provided by the Centro de Hemoterapia y Hemodonación de Castilla y León (Valladolid, Spain). Written informed consent was obtained from each donor. The buffy coats were diluted 1:1 with phosphate-buffered saline (PBS), and the mononuclear cells were collected by centrifugation at 750× *g* for 30 min over a Ficoll-Paque cushion. The mononuclear cell ring was recovered, washed three times with PBS, and allowed to adhere on sterile dishes for 2 h at 37 °C. Nonadherent cells were removed by washing extensively with PBS. The adhered monocytes were placed in RPMI 1640 medium (Molecular Probes-Invitrogen, Carlsbad, CA, USA) supplemented with 40 µg/mL gentamicin, and used the following day [35,36]. All experiments were conducted in serum-free media. For the preparation of zymosan, the particles were suspended in PBS, boiled for 60 min, and washed three times. The final pellet was resuspended in PBS at 20 mg/mL, and stored frozen. Zymosan aliquots were diluted in serum-free medium and sonicated before addition to the cells [37]. To obtain opsonized zymosan, the particles were treated with heat-inactivated human serum (3 mg zymosan per mL serum) for 20 min at 37 °C. No phospholipase A_2_ activity was detected in the zymosan batches used in this study, as assessed by in vitro assay under different conditions [38,39,40]. Cell protein content was quantified according to Bradford [41], using a commercial kit (BioRad Protein Assay, Bio-Rad, Hercules, CA, USA).

### 2.3. Measurement of POA Incorporation into Phospholipids and Phospholipid Remodeling

The cells were exposed to [9,10-^3^H]POA (0.25 µCi/mL) for different periods of time. Total lipids in the cell monolayers were extracted according to Bligh and Dyer [42]. The lipids were separated by thin-layer chromatography using n-hexane/diethyl ether/acetic acid (70:30:1, *v*/*v*/*v*) as the mobile phase [43]. The phospholipid classes were separated by thin-layer chromatography, using plates impregnated with boric acid [44], and the process was run twice. The mobile phase consisted of chloroform/methanol/28% (*w*/*w*) ammonium hydroxide (60:37.5:4, *v*/*v*/*v*) [45,46]. The different bands were scraped from the plates and their radioactive content was determined by scintillation counting using a LS 6500 counter (Beckman Coulter, Brea, CA, USA). For the analysis of phospholipid fatty acid remodeling, the cells were pulse-labeled with 1 nM [^3^H]POA (0.25 μCi/mL) for 30 min at 37 °C. The cells were then washed with medium containing 0.5 mg/mL bovine serum albumin to remove the non-incorporated label. Afterward, the cells were placed in serum-free medium and incubated at 37 °C for the indicated periods of time. After lipid extraction, the phospholipid classes were separated by thin-layer chromatography, as indicated above. The spots corresponding to each phospholipid class were cut out and assayed for radioactivity by liquid scintillation counting [47,48].

### 2.4. Gas Chromatography–Mass Spectrometry (GC-MS) Analyses

Cell samples (approx. 10^7^ cells) were washed with PBS, lysed with water, and sonicated in a tip homogenizer twice for 15 s prior to total lipid extraction, according to Bligh and Dyer [42]. The following internal standard was added before lipid extraction: 10 nmol of 1,2-diheptadecanoyl-sn-glycero-3-phosphocholine. Lipid classes were separated by thin-layer chromatography, as described in the preceding paragraph. The bands corresponding to the different phospholipid classes were scraped from the plate, and fatty acid methyl esters were obtained by transmethylation with 500 µL of 0.5 M KOH in methanol for 60 min at 37 °C [49,50]. Then, 500 µL of HCL was added to neutralize, and fatty acid methyl esters were extracted twice with 500 µL of n-hexane. The analysis of fatty acid methyl esters was carried out using an Agilent 6890 N gas chromatograph coupled to an Agilent 5975 mass-selective detector operated in electron impact mode (EI, 70 eV), equipped with an Agilent DB23 column (60 m × 0.25 mm internal diameter × 0.15 μm film thickness) (Agilent Technologies, Santa Clara, CA, USA) [49,50]. Then, 1 μL of sample was injected in splitless mode. The inlet temperature was maintained at 250 °C. The oven temperature was held at 50 °C for 1 min, and then increased to 175 °C at intervals of 25 °C per min, and to 230 °C at intervals of 2.75 °C per min. The final temperature was maintained for 5 min, and the run time was 33 min. The mass spectrometry transfer line was maintained at 250 °C and the mass spectrometer quadrupole and source at 150 °C and 230 °C, respectively. Helium was used as a carrier gas at a constant pressure of 180 kPa. Data acquisition was carried out in both scan and selected ion monitoring modes. Scan mode was used for compound identification. This was accomplished by comparing with authentic standards and the National Institute of Standards and Technology Mass Spectrometry library spectra (Gaithersburg, MD, USA). Selected ion monitoring mode was used for quantitation, using 74 and 87 fragments for saturated, 83 for monounsaturated, 67 and 81 for diunsaturated and 79 and 91 for polyunsaturated fatty acid methyl esters. A 37-component mixture was used for calibration curves (Supelco Inc., Bellefonte, PA, USA), and nonadecanoic acid was used as an internal standard. Data analysis was carried out with the Agilent G1701EA MSD Productivity Chemstation software, revision E.02.00.

### 2.5. Liquid Chromatography/Mass Spectrometry (LC-MS) Analyses

Lipids corresponding to 50 µg of cell homogenate protein were extracted according to Bligh and Dyer [42], and the following internal standards were added: 20 pmol each of 1,2-dimyristoyl-sn-glycero-3-phosphoglycerol, 1,2-dilauroyl-sn-glycero-3-phosphoethanolamine, 1,2-dimyristoyl-sn-glycero-3-phosphoethanolamine, 1,2-diheptadecanoyl-sn-glycero-3-phosphoethanolamine, 1,2-dimyristoyl-sn-glycero-3-phosphoserine, 1,2-dimyristoyl-sn-glycero-3-phosphate, 1,2-dipentadecanoyl-sn-glycero-3-phosphocholine, 1,2-diheptadecanoyl-sn-glycero-3-phosphocholine, and 1,2-dinonadecanoyl-sn-glycero-phosphocholine. The samples were re-dissolved in hexanes/2-propanol/water (42:56:2, *v*/*v*/*v*) and injected into a Thermo Fisher Scientific Dionex Ultimate 3000 high-performance liquid chromatograph equipped with an Ultimate HPG-3400SD standard binary pump and an Ultimate ACC-3000 autosampler column compartment (Thermo Fisher Scientific, Waltham, MA, USA). Separation was carried out using a FORTIS HILIC (150 × 3 mm, 3 µm particle size) (Fortis Technologies, Neston, UK). The mobile phase consisted of a gradient of solvent A (hexanes/isopropanol, 30:40, by volume) and solvent B (hexanes/isopropanol/20 mM ammonium acetate in water, 30:40:7, *v*/*v*/*v*). The gradient started at 75% A from 0 to 5 min, then decreased from 75% A to 40% A at 15 min and from 40% A to 5% A at 20 min, then held at 5% until 40 min, and increased to 75% at 41 min. Then, the column was re-equilibrated, holding at 75% A for an additional 14 min period before the next sample injection [51]. Phospholipid species were analyzed in scheduled multiple reaction monitoring mode with negative ionization. All glycerophospholipids were detected as [M-H]^–^ ions except choline glycerophospholipids, which were detected as [M + CH_3_COO]^−^ ions. Quantification was carried out by integrating the chromatographic peaks of each species and comparing with the peak area of the internal standards that corresponded to each class. The flow rate through the column was fixed at 0.4 mL/min. The liquid chromatography system was coupled online to an AB Sciex QTRAP 4500 mass spectrometer equipped with a Turbo V ion source and a TurbolonSpray probe for electrospray ionization (AB Sciex, Framingham, MA, USA). Source parameters were set as follows: ion spray voltage, −4500 V; curtain gas, 30 psi; nebulizer gas, 50 psi; desolvation gas, 60 psi; temperature, 425 °C. Phospholipid species were analyzed in scheduled multiple-reaction monitoring mode with negative ionization, detecting the *m*/*z* of 253.2 in Q3, corresponding to palmitoleic acid as [M-H]–. Compound parameters were fixed as follows: declustering potential: −45 V (PC), −60 V (PE), −30 V (PI), −50 V (PS), −60 V (phosphatidic acid), −50 V (phosphatidylglycerol); collision energy: −50 V (PC), −40 V (PE), −60 V (PI), −50 V (PS), −45 V (phosphatidic acid), −45 V (phosphatidylglycerol); entrance potential: −10 V; and collision cell exit potential: −8 V. All phospholipid species were detected as [M-H]^−^ ions except the PC species, which were detected as [M + CH_3_COO]^−^ ions. Quantification was carried out by integrating the chromatographic peaks of each species and comparing with the peak area of the internal standard that corresponded to each class [29,52].

### 2.6. Statistical Analysis

The results are shown as mean ± standard error of the mean, and correspond to at least three independent determinations with incubations in duplicate. For statistical significance the data were analyzed with a *t*-test (two groups) or ANOVA (more than two groups), followed by Tukey’s post hoc test, using SigmaPlot software, version 14.0 (Systat Software Inc., San Jose, CA, USA). A value of *p* < 0.05 was considered statistically significant.

## 3. Results

**Figure 1:**
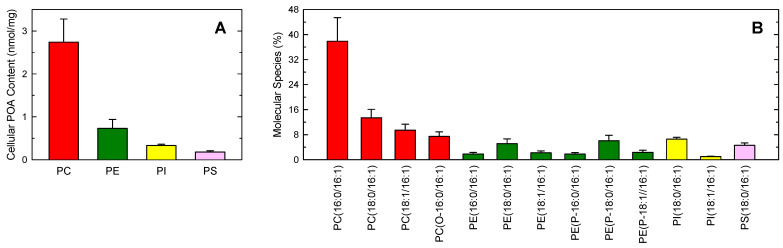
Phospholipid distribution of POA in human monocytes. (**A**) Cellular POA content in phospholipids as shown by class. The different phospholipid classes were separated by thin-layer chromatography and their POA content was determined by GC-MS after converting the fatty acid glyceryl esters into fatty acid methyl esters. (**B**) Profile of POA-containing phospholipid molecular species. The distribution profile of POA between phosphatidylcholine (PC, red bars), phosphatidylethanolamine (PE, green bars), phosphatidylinositol (PI, yellow bars), and phosphatidylserine (PS, pink bars) species was determined by LC-MS. The data are shown as mean values ± standard error (n = 3).

Previous work from our laboratory has demonstrated the presence of significant amounts of POA in human monocytes, as well as its biological relevance as an anti-inflammatory lipid signal [25,26]. Figure 1A shows the mass distribution of POA among the various phospholipid classes of human monocytes, as measured by GC-MS. PC contained most of the POA present in these cells, amounting to approximately 70% of total cellular fatty acid. The POA content in other phospholipid classes was much lower. Next, the distribution of POA among the different phospholipid molecular species was determined by LC-MS, and the results are shown in Figure 1B. In these experiments, the molecular species of interest were unequivocally identified by multiple reaction monitoring experiments, where the fragment of *m*/*z* 253.2, which corresponds to the [M-H]^−^ of the fatty acid, was detected in Q3. Following nomenclature recommendations, fatty chains within phospholipids are designated by their number of carbons:double bonds. A designation of O- before the first fatty chain indicates that the sn-1 position is ether-linked, whereas a P- designation indicates a plasmalogen form (sn-1 vinyl ether linkage) [53,54,55]. One single species, namely PC(16:0/16:1), contained almost one half of all cellular fatty acid (Figure 1B). All other major species were of the PC class. Not even one molecular species from the other classes (i.e., PE, PI, or PS) comprised more than 7% of total cellular fatty acid.

**Figure 2:**
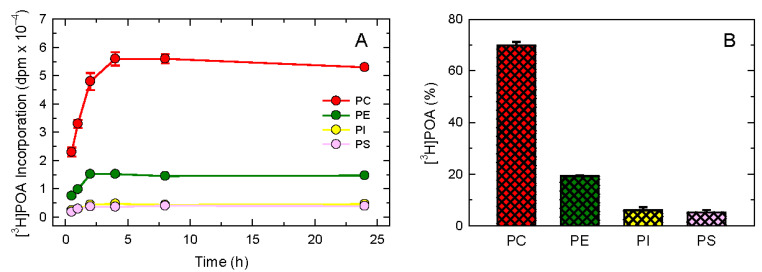
Characterization of POA incorporation into macrophage phospholipids. (**A**) Time-course of incorporation into different phospholipid classes. The cells were incubated with [^3^H]POA for the indicated periods of time. Lipids were then extracted, and [^3^H]POA incorporation was measured in PC (red), PE (green), PI (yellow), and PS (pink). (**B**) The cells were exposed to [^3^H]POA for 30 min, thoroughly washed to eliminate the unincorporated label, and left in culture for 24 h. Afterward, the [^3^H]POA content in each phospholipid class was measured. The data are shown as mean values ± standard error (n = 3).

This propensity of POA to incorporate into choline-containing molecular species is striking and prompted us to characterize further the cellular mechanisms of phospholipid incorporation of POA in human monocytes, as well as the possible existence of fatty acid remodeling reactions between phospholipids. For these experiments, we used tritium-labeled POA, which improved sensitivity. Figure 2A shows that the exposure of the human monocytes to exogenous [^3^H]POA resulted in the rapid incorporation of the fatty acid into the different phospholipid classes. Consistent with its endogenous mass distribution, shown in Figure 1, [^3^H]POA incorporated primarily into PC, followed by PE, PI, and PS.

It is remarkable that in Figure 2A, the ratio of incorporation of the fatty acid into the various phospholipid classes was preserved at all time points, i.e., PC accounted for 60–70% of total fatty acid incorporation at all times tested, and the percent of label incorporated into PE, PI, and PS also remained the same at all times. These results suggest that, once incorporated into cellular phospholipids, POA is not further transferred between phospholipid classes by the actions of acyltransferases or transacylases. Thus, cells labeled for 24 h distribute the POA into phospholipid classes in a manner that represents the mass distribution (*cf*. Figure 1A and Figure 2A).

To confirm these findings, we conducted a pulse experiment where the monocytes were exposed to [^3^H]POA for 30 min, thoroughly washed to remove any unincorporated label, and then left in culture free of labeled fatty acid. Under these conditions, the relative distribution of the label between phospholipid classes was measured. Figure 2B shows that such a distribution did not vary with time; the fatty acid that was initially incorporated into either PC, PE, PI, or PS remained in its initial location. Thus, the distribution of POA was almost identical to that determined during the time-course experiment depicted in Figure 2A, and also very similar to that found for the endogenous preexisting fatty acid mass (Figure 1A). Collectively, these results suggest that the rate of incorporation of POA into the different classes of phospholipids does not change with time, and the fatty acid remains in the phospholipid class that initially incorporated it. This behavior contrasts with that of polyunsaturated fatty acids such as arachidonic acid (AA) [56,57,58,59], which are known to undergo profound remodeling between phospholipids that substantially changes their initial distribution [60,61,62]. 

**Figure 3:**
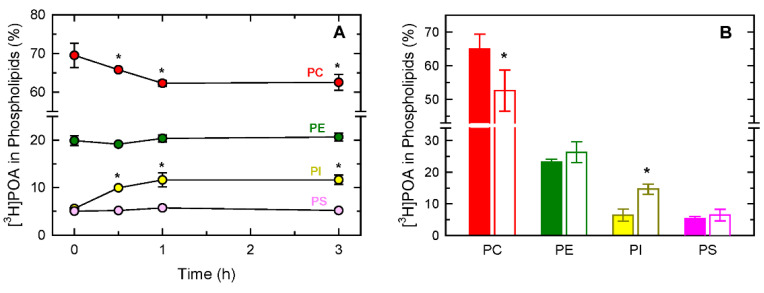
Effect of monocyte activation on the distribution of POA into the different phospholipid classes. The monocytes were exposed to [^3^H]POA for 30 min, thoroughly washed to eliminate the unincorporated label, and left in culture in the presence of 1 mg/mL zymosan for the indicated periods of time (**A**), or 1 µM calcium ionophore A23187 for 3 h (**B**) (unstimulated cell conditions are shown in solid bars; A23187-treated cell conditions are shown in open bars). Afterward, the [^3^H]POA content in each phospholipid class was measured. The data are shown as mean values ± standard error (n = 3). * *p* < 0.05, significantly different from the corresponding species in control unstimulated cells (time zero in (**A**); solid bars in (**B**)).

In the next series of experiments, we investigated whether the cellular distribution of POA was affected by the activation state of the cells. For this purpose, we designed an experiment similar to the one described in Figure 2B, but in this case, after the initial labeling, the cells were exposed to yeast-derived zymosan. This compound has long been used as a model stimulus for the study of pathways involving fatty acid mobilization in monocytes and macrophages [63,64,65,66,67,68]. Zymosan induced a time-dependent decrease in the amount of [^3^H]POA in PC that was somewhat paralleled by an increase in the fatty acid in PI. No changes were detected in PE or PS (Figure 3A). To extend these observations to other monocyte activation conditions, these experiments were repeated utilizing the calcium ionophore A23187 as a stimulus. This compound is also widely used for studies assessing phospholipid fatty acid turnover in phagocytic cells [69,70,71,72,73]. The data in Figure 3B showed that the ionophore also reduced the content of POA in PC, while increasing it in PI. 

**Figure 4:**
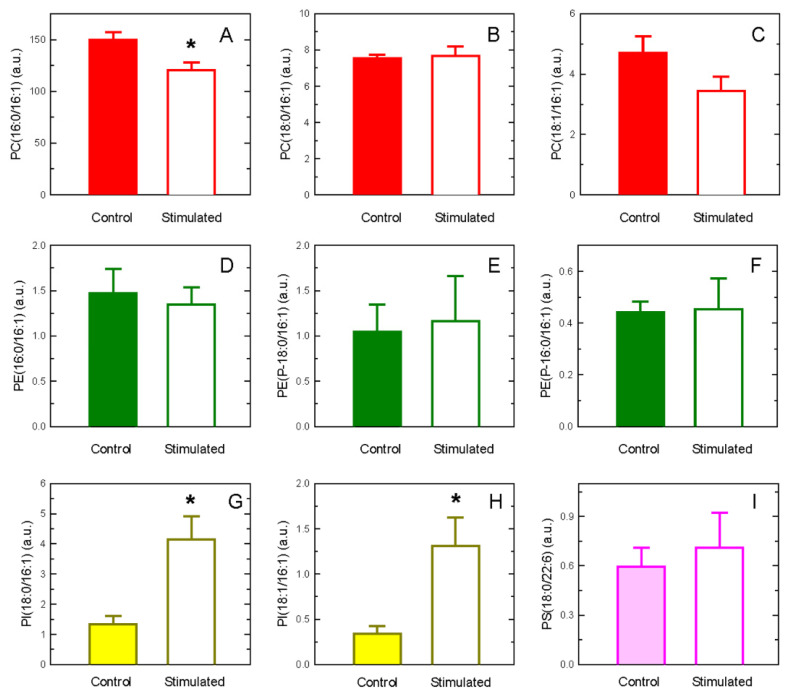
Changes in POA-containing species in human monocytes. The levels of major POA-containing PC (**A**–**C**), PE (**D**–**F**), PI (**G**,**H**), and PS (**I**) molecular species in resting (Control) and stimulated monocytes were determined by LC-MS. The data are shown as mean values ± standard error (n = 3). * *p* < 0.05, significantly different from the corresponding species in control cells. a.u., arbitrary units.

LC-MS analyses of POA-containing phospholipid molecular species revealed significant decreases in the POA content of the major molecular species PC(16:0/16:1) in activated cells (Figure 4). A tendency for the minor species PC(18:1/16:1) to decrease was noted as well, but the difference failed to reach significance. On the other hand, two PI species whose POA levels increased after cell stimulation were clearly identified, i.e., PI(18:0/16:1) and PI(18:1/16:1) (Figure 4). In agreement with the data shown in Figure 3, no changes in the POA content of PE and PS could be detected (Figure 4).

**Figure 5:**
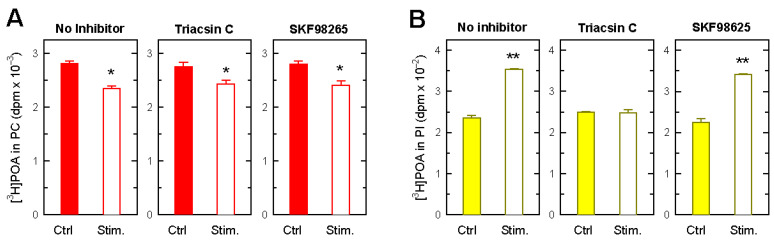
Effect of triacsin C and SKF98625 on the movement of POA between phospholipids in zymosan-stimulated monocytes. The [^3^H]POA-prelabeled cells were either unstimulated (Ctrl, colored bars) or stimulated by 1 mg/mL zymosan for 3 h (Stim, open bars) in the absence (No inhibitor) or presence of 3 µM triacsin C or 10 µM SKF98625, as indicated. Afterward, ^3^H-radioactivity levels in PC (**A**) and PI (**B**) were measured. The data are shown as mean values ± standard error (n = 3). * *p* < 0.05, ** *p* < 0.01, significantly different from control (Ctrl).

To determine the cellular mechanisms underlying the changes in POA levels in PC and PI during cell activation, experiments using [^3^H]POA-labeled cells were conducted with triacsin C, an inhibitor of long-chain acyl-CoA synthetases [74,75,76]. Human monocytes are known to express all five forms of mammalian long-chain acyl-CoA synthetases, namely ACSL-1, -3, -4, -5, and -6 [30]. Of these, the triacsin C-sensitive forms are ACSL-1, -3, and -4, and previous studies have demonstrated that these are the major isoforms involved in the CoA-dependent regulation of fatty acid incorporation into phospholipids [77]. Notably, Figure 5 shows that triacsin C completely blocked the increased formation of POA-containing PI in the activated monocytes. Note, as well, that the decrease in POA levels in PC occurring as a consequence of monocyte activation was not blunted by triacsin C, suggesting that the release of POA from PC is due to the action of a bona fide phospholipase. In parallel experiments, we also tested the effect of SKF98625, an inhibitor of the CoA-independent transfer of polyunsaturated fatty acids between phospholipids in various cell types [78,79]. This compound exerted no discernible effects on POA levels in either PC or PI in the activated cells, as compared to the untreated cells. 

Because POA has been shown to localize preferentially to the sn-2 position of the glycerol moiety of PC species in phagocytic cells [57], it appears logical to suggest that the phospholipase(s) involved in the liberation of POA from PC in monocytes is/are of the A_2_ type. Accordingly, in the next set of experiments we used a number of well-established inhibitors of the major cellular PLA_2_ forms potentially involved in fatty acid mobilization in activated cells. To inhibit the group IVA cytosolic phospholipase A_2_α (cPLA_2_α) we used pyrrophenone. This inhibitor potently and selectively blocks cPLA_2_α in cells without significantly affecting the activities of the other cellular phospholipase A_2_s [80,81,82]. To inhibit the activity of the multiple secreted phospholipase A_2_ (sPLA_2_) enzymes present in human leukocytes that are potentially capable of effecting the fatty acid release [83], we used the pan-sPLA_2_ inhibitor LY311727, an indomethacin analogue that blocks this kind of enzymes without exerting significant effects on other cellular phospholipase A_2_s [84,85,86]. To inhibit the group IVA calcium-independent PLA_2_ (iPLA_2_β), we used FKGK18, a fluoroketone that selectively inhibits iPLA_2_β in cells at concentrations at least 200-fold lower than those required to have an effect on either cPLA_2_α or sPLA_2_ [87,88]. For comparative purposes, we also used the structurally unrelated inhibitor bromoenol lactone (BEL) to inhibit cellular iPLA_2_β. This compound inhibits cellular calcium-independent PLA_2_ activity at concentrations at which calcium-dependent enzymes are not affected, although it may exert off-target effects depending on cell type and stimulation conditions [89,90,91]. 

**Figure 6:**
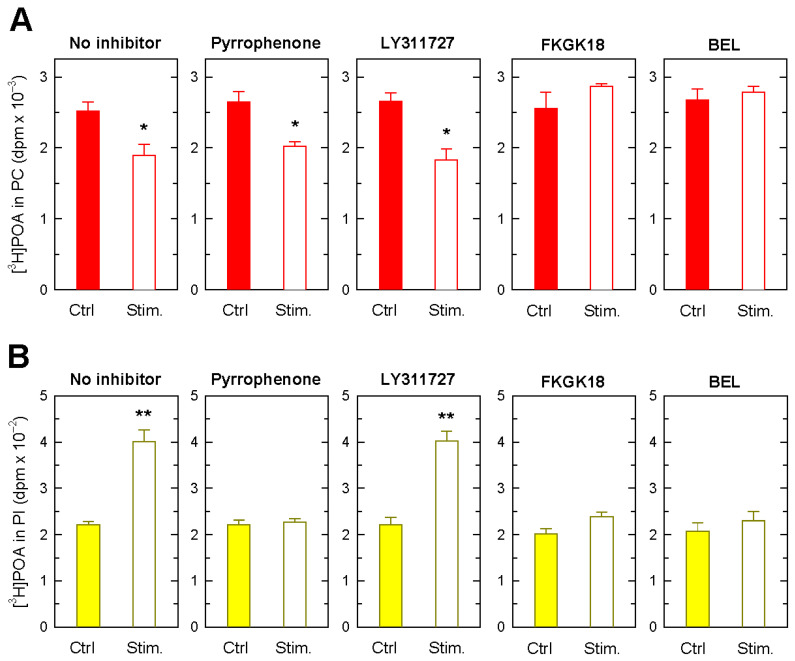
Effect of phospholipase A_2_ inhibitors on the movement of POA between phospholipids in zymosan-stimulated monocytes. The [^3^H]POA-prelabeled cells were either unstimulated (Ctrl, colored bars) or stimulated by 1 mg/mL zymosan for 3 h (Stim, open bars) in the absence (No inhibitor) or presence of the following inhibitors: 1 µM pyrrophenone, 25 µM LY311727, 10 µM FKGK18, or 10 µM BEL, as indicated. Afterward, ^3^H-radioactivity levels in PC (**A**) and PI (**B**) were measured. The data are shown as mean values ± standard error (n = 3). * *p* < 0.05, ** *p* < 0.01, significantly different from control (Ctrl).

Figure 6A shows that the inhibitors exerted disparate effects on the levels of POA in PC; while LY311727 had no discernible effect, both FKGK18 and BEL completely abrogated the zymosan-induced decrease in POA levels in PC. Consistent with these data, LY311727 had no effect on the zymosan-induced increases in POA levels in PI, while both FKGK18 and BEL completely blocked such increases. Collectively, these results indicate that sPLA_2_ enzymes do not participate in the stimulus-induced transfer of POA moieties for PC to PI. On the opposite side, the inhibition of iPLA_2_β results in the complete inhibition of POA repositioning between PC and PI in the activated cells, suggesting that this is the phospholipase that removes the POA from PC in order for it to be incorporated into PI later via CoA-dependent acyltransferases.

Of note, pyrrophenone treatment of the cells had no effect on the zymosan-induced POA decreases in PC (Figure 6A), yet the inhibitor completely blocked the stimulated formation of POA-containing PI (Figure 6B). These data suggest that there is a cPLA_2_α-sensitive step in the stimulated formation of POA-containing PI that is not related to the zymosan-induced changes in POA-containing PC.

**Figure 7:**
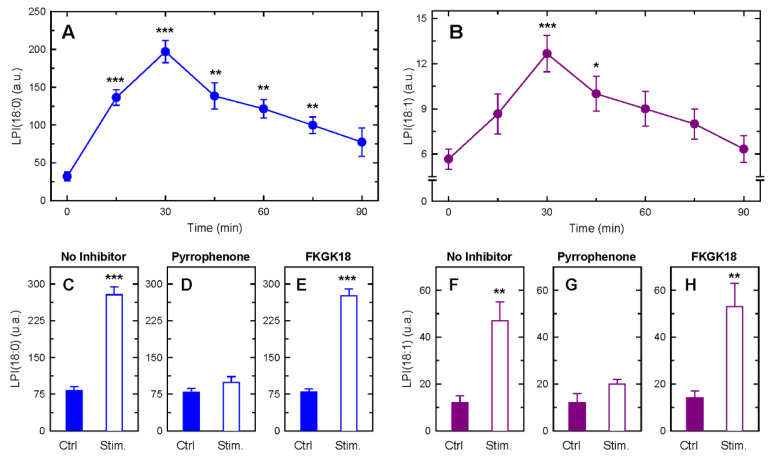
LysoPI production by zymosan-stimulated human monocytes. Time-course of the zymosan-stimulated formation of LPI(18:0) (**A**) and LPI(18:1) (**B**), as measured by LC-MS. These data are shown as mean values ± standard error (n = 3). * *p* < 0.05, ** *p* < 0.01, *** *p* < 0.001 represent significant difference from unstimulated cells at time zero. To assess the effect of phospholipase A_2_ inhibitors on lysoPI formation, the cells were pretreated for 15 min with 1 µM pyrrophenone (**D**,**G**), 1 µM FKGK18 (**E**,**H**) or neither (labeled as ‘No inhibitor’) (**C**,**F**), as indicated. Afterward, the cells were stimulated with zymosan, and LPI(18:0) (**C**–**E**) or LPI(18:1) (**F**–**H**) were measured by LC-MS. These data are shown as mean values ± standard error (n = 3). ** *p* < 0.01, *** *p* < 0.001, significantly different from control (Ctrl).

Given that PI is particularly enriched with AA in phagocytic cells, including human monocytes [56,57,58], we reasoned that the stimulated formation of lysoPI that occurs as a by-product of the stimulus-induced cPLA_2_α-mediated AA mobilization [77,92,93] could represent the aforementioned step. In accordance with this view, zymosan-stimulated monocytes exhibited a time-dependent accumulation of the lysoPI molecular species LPI(18:0) and LPI(18:1), i.e., the two species incorporating elevated amounts of POA during zymosan stimulation (Figure 7A,B). Such an elevation was blunted by pyrrophenone (Figure 7D,G) but not by FKGK18 (Figure 7E,H).

## 4. Discussion

The present study examined the incorporation and remodeling of POA between phospholipid pools during the activation of human peripheral blood monocytes. In resting monocytes, POA incorporates primarily into PC, followed by PE, PI, and PS. This distribution does not change with time, i.e., the POA remains in the phospholipid class that initially incorporated it. Importantly, however, phospholipid fatty acid remodeling involving this fatty acid does occur after the cells are stimulated by either receptor-directed (zymosan) or soluble (calcium ionophore) agonists. Thus, there is a stimulated movement of POA moieties from PC to PI. While this transfer does not drastically change the distribution of POA among cellular phospholipids, i.e., PC still remains as the major POA-containing phospholipid in the cells, there is an enrichment with POA of two defined molecular species of PI, namely PI(18:0/16:1) and PI(18:1/16:1). Such enrichments increase the cellular mass of PI(18:0/16:1) and PI(18:1/16:1) by about 3.1- and 3.8-fold, respectively. Thus, it seems likely that these two species may play significant regulatory roles during the execution of cellular responses by the activated monocytes. Clearly, future work should be devoted to identifying cellular responses that imply these PI species, so that they can be included in the growing number of phospholipid molecular species with specific roles in stimulus–response coupling [94,95,96,97,98,99]. While addressing these cellular responses is beyond the scope of the current work, it is worth mentioning here that at least one of these species, namely PI(18:0/16:1), was described to possess growth-factor-like activity on fibroblasts [99]. More recently, the related species, PI(18:1/18:1), although lacking POA, was described to link the metabolism of unsaturated fatty acids with stress signaling [100].

**Figure 8:**
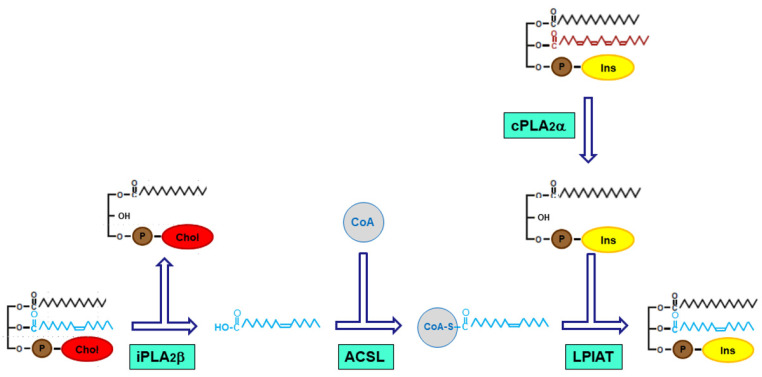
Proposed pathway for the movement of palmitoleic acid from PC to PI in human monocytes. For simplicity, the figure shows the formation of PI(18:0/16:1). Formation of PI(18:1/16:1) would proceed following the same steps when the cPLA_2_α acts upon PI(18:1/20:4) instead of PI(18:0/20:4).

Examination of the metabolic pathway regulating the increased formation of POA-containing molecular species in activated monocytes reveals that it proceeds through fatty acid recycling via phospholipase A_2_/CoA-dependent acyltransferases, i.e., the Lands cycle [101,102], rather than via CoA-independent, direct transfer of POA moieties between phospholipids, as occurs predominantly when the fatty acid to be remodeled is a polyunsaturate such as AA [60,61,62]. Importantly, the pathway appears to utilize two different phospholipase A_2_ enzymes; the first one provides the free fatty acid, and the second one provides the lysoPI acceptor (Figure 8). Thus, this route constitutes a prime example of the interaction of different phospholipase A_2_ enzymes, acting separately but in concert, within lipid signaling pathways. Utilizing different substrates, these phospholipase A_2_s contribute to generate lipid diversity, which may conceivably help the cell to respond properly to stimuli.

According to this pathway (Figure 8), POA is mobilized primarily from 1-palmitoyl-2-palmitoleoyl-glycero-3-phosphocholine (PC(16:0/16:1)) by iPLA_2_β. In parallel, cPLA_2_α hydrolyzes the arachidonate-containing PI species 1-stearoyl-2-arachidonoyl-glycero-3-phosphoinositol (PI(18:0/20:4)) and 1-oleoyl-2-arachidonoyl-glycero-3-phosphoinositol (PI(18:1/20:4)) to produce the corresponding lysophospholipids, LPI(18:0) and LPI(18:1). Acyl-CoA synthetases (ACSL) generate palmitoleoyl-CoA, which will be used by CoA-dependent acyltransferases (LPIAT) to form PI(18:0/16:1) and PI(18:1/16:1). 

Although the movement of POA between PC and PI appears to be a quantitatively important process, it is also certainly possible that other reactions may also occur at the same time. Our earlier studies showed that POA tends to accumulate in neutral lipids when the monocytes transition into foamy cells [30] and, in murine peritoneal macrophages, POA is used for the formation of the anti-inflammatory branched ester palmitoleyl hydroxystearate [29]. We have not detected these reactions in the human monocytes, which is probably due, on one hand, to the fact that human monocytes contain lesser amounts of POA than murine peritoneal macrophages [26] and, on the other hand, to cell type differences arising from specialized roles in physiology and pathophysiology.

The involvement of iPLA_2_β as the initiating enzyme in the pathway leading to the elevated formation of POA-enriched PI species is of special interest. Unlike other members of the phospholipase A_2_ superfamily, such as the sPLA_2_s or cPLA_2_α, iPLA_2_β appears to lack an ‘activating switch’ that increases its cellular activity after stimulation, either by increasing the enzyme mass or the intrinsic enzyme activity [89,90,91]. While iPLA_2_β manifests no evident substrate preference when its activity is determined in in vitro assays [103,104,105], studies with primary cell cultures, iPLA_2_β-overexpressing cells, or cells from iPLA_2_β knock-out animals have established that iPLA_2_β manifests in cells a clear preference for hydrolyzing PC species containing palmitic acid at the sn-1 position [57,106,107,108,109,110]. This is in perfect agreement with the data of this study, and aligns with our hypothesis that the substrate specificities of phospholipase A_2_ enzymes, as observed in vivo, may be constrained by their subcellular localization [111]. Substrate compartmentalization may therefore constitute a major mechanism for the cellular regulation of iPLA_2_β activity on membrane phospholipids [111].

While a large number of studies on phospholipid fatty acid remodeling have focused on the exchange of polyunsaturated fatty acids mainly between PC and PE [60,61,62,77], it seems likely that PI species may also actively participate in lipid membrane modulation. Thus, an intriguing possibility that stems from the data presented in this study is whether fatty acid transfer from PC to PI is bidirectional, i.e., whether part of the AA released from PI by cPLA_2_α in the activated cells may be reacylated into PC to compensate, at least in part, the POA lost from PC through the action of iPLA_2_β. This attractive possibility cannot be directly tested in a study such as this one, which deals with live cells, because it is not possible to distinguish the PI-bound AA from the same fatty acid that is already present in PC which, to compound, represents a larger pool [112]. Studies with different cell types such as human neutrophils or murine macrophages have provided support to the concept that specific phospholipid pools are linked to the formation of particular eicosanoids, with PC likely playing a major role [57,59,113,114,115,116]. In addition, it is worth remarking that zymosan-stimulated AA mobilization in human monocytes, which involves the hydrolysis of both PC and PI, is not altered by the BEL inhibition of iPLA_2_β [35]. The latter condition is shown in this study to result in the diminished mobilization of POA from PC.

## 5. Conclusions

In this work, we show that remodeling of POA between phospholipid molecular species occurs in activated human monocytes. The process, which is CoA-dependent, involves the participation of two different intracellular phospholipase A_2_s acting in concert but separately to provide the free POA, on one hand, and the lysophospholipid acceptor on the other. By using mass spectrometry, we identified the major provider of POA as the molecular species PC(16:0/16:1), and two PI molecular species, namely PI(18:0/16:1) and PI(18:1/16:1), as major products within this remodeling pathway. It seems likely that accumulation of POA in defined PI species during the activation of human monocytes may underlie some of the biological effects attributed to this fatty acid.

## Data Availability

Data are contained within the article.

## Abbreviations

POA	palmitoleic acid (cis-9-hexadecenoic acid, 16:1n–7)
AA	arachidonic acid (cis-5,8,11,14-eicosatetraenoic acid, 20:4n–6)
PC	phosphatidylcholine
PE	phosphatidylethanolamine
PI	phosphatidylinositol
PS	phosphatidylserine
cPLA_2_α	group IVA cytosolic phospholipase A_2_α
iPLA_2_β	group VIA calcium-independent phospholipase A_2_
sPLA_2_	secreted phospholipase A_2_
